# Reporting delays: A widely neglected impact factor in COVID-19 forecasts

**DOI:** 10.1093/pnasnexus/pgae204

**Published:** 2024-05-22

**Authors:** Long Ma, Zhihao Qiu, Piet Van Mieghem, Maksim Kitsak

**Affiliations:** Faculty of Electrical Engineering, Mathematics, and Computer Science, Delft University of Technology, Delft, GA 2600, The Netherlands; Faculty of Electrical Engineering, Mathematics, and Computer Science, Delft University of Technology, Delft, GA 2600, The Netherlands; Faculty of Electrical Engineering, Mathematics, and Computer Science, Delft University of Technology, Delft, GA 2600, The Netherlands; Faculty of Electrical Engineering, Mathematics, and Computer Science, Delft University of Technology, Delft, GA 2600, The Netherlands

**Keywords:** COVID-19 pandemic, reporting delays, epidemic forecasts, parametric optimization, SIRD compartmental model, time series analysis

## Abstract

Epidemic forecasts are only as good as the accuracy of epidemic measurements. Is epidemic data, particularly COVID-19 epidemic data, clean, and devoid of noise? The complexity and variability inherent in data collection and reporting suggest otherwise. While we cannot evaluate the integrity of the COVID-19 epidemic data in a holistic fashion, we can assess the data for the presence of reporting delays. In our work, through the analysis of the first COVID-19 wave, we find substantial reporting delays in the published epidemic data. Motivated by the desire to enhance epidemic forecasts, we develop a statistical framework to detect, uncover, and remove reporting delays in the infectious, recovered, and deceased epidemic time series. Using our framework, we expose and analyze reporting delays in eight regions significantly affected by the first COVID-19 wave. Further, we demonstrate that removing reporting delays from epidemic data by using our statistical framework may decrease the error in epidemic forecasts. While our statistical framework can be used in combination with any epidemic forecast method that intakes infectious, recovered, and deceased data, to make a basic assessment, we employed the classical SIRD epidemic model. Our results indicate that the removal of reporting delays from the epidemic data may decrease the forecast error by up to 50%. We anticipate that our framework will be indispensable in the analysis of novel COVID-19 strains and other existing or novel infectious diseases.

**Significance Statement**

Accurate forecasts constitute the first step toward controlling an epidemic outbreak. However, the accuracy of such forecasts strongly depends on input data. Here, we develop a statistical framework to identify and remove reporting delays from the epidemic data. We use this framework to de-noise epidemic data of the first COVID-19 wave in eight regions worldwide. We demonstrate that the removal of reporting delays may increase the accuracy of epidemic forecasts by up to 50%. We anticipate that our framework will be invaluable in the analysis and forecasts of existing and future epidemics.

## Introduction

The COVID-19 pandemic has been crippling the world’s health, economies, and quality of life for over 3 years. We are gradually understanding that COVID-19 is here to stay. Fast mutation rates of the virus and its overwhelming spreading capability make it extremely hard, if not impossible, to eradicate (1). COVID-19 is not the first and almost surely not the last pandemic to hit humanity. Therefore, in order to better prepare and withstand other contagious diseases in the future, we need to extract as many lessons as possible from the COVID-19 pandemic.

Public awareness is, arguably, the first line of defense against any infectious disease. Efficient collection of epidemic data and accurate epidemic forecasts allow for timely containment of the spread or *flattening of the curve* to win time for the development of pharmaceutical treatment methods. Due to the success of network epidemiology and the broad availability of data, significant advances in epidemic modeling and forecast methods (2–6) have been achieved. However, the accuracy of epidemic forecasts strongly depends on the accuracy and timeliness of the input data.

Are epidemic data—especially in the short-time period after the onset of the epidemic—devoid of inaccuracies? Multiple sources suggest the negative answer. Indeed, public health indicators are known to be biased, because they are sensitive to fluctuations in supply and demand for diagnostic testing (7–9). Likewise, inequalities in geographic accessibility have been documented to adversely affect the coverage and timelines of health indicators (10, 11). It is now well understood that ignoring epidemic data inaccuracies may lead to delays in deploying intervention policies resulting in dire consequences of an epidemic on the population’s health (12, 13).

One prominent challenge is the existence of temporal delays in data reporting (14, 15), defined as the time difference between the event when the person was affected by the virus and the time this event is accounted for. The reporting delays may occur due to many reasons, including patient hesitancy, medical testing delays, and reporting delays by public health authorities (16, 17). Consequently, reporting delays may vary not only across different diseases but also across different regions of interest. The median diagnosis delay for malaria is, for instance, approximately 4 days (18). Similarly, the research on the Middle East respiratory syndrome coronavirus (MERS-CoV) found a time difference between the symptom onset and confirmation of approximately four days (19). Hepatitis A, measles, and mumps data are usually reported after eight days (16). Reporting delays for some diseases are significantly longer: Hepatitis B, Shigellosis, and Salmonella can take on average 2–3 weeks (16). Recent COVID-19 studies reveal significant reporting delays of infections in China (20–25), Italy (26), Germany (27, 28), Singapore (29), the United States of America (30), and the United Kingdom (14).

While there is a plethora of scientific works aimed at the reconstruction of network data, including the reconstruction of network data for epidemic forecasts (31–33), we lack inference methods to assess and improve the accuracy of epidemic data itself. Instead, the prevalent epidemic forecast philosophy is focused either on data fusion (34–36)—or machine learning methods (37–39).

In our work, we develop a statistical framework to remove reporting delays. We demonstrate that the removal of reporting delays may significantly improve the accuracy of epidemic forecasts. Our statistical framework can be used in combination with any epidemic forecast method that takes infectious, recovered, and deceased epidemic data as input. Our work is organized as follows. We first present the evidence for the presence of reporting delays in the epidemic data extracted from the first COVID-19 wave. We then proceed to develop a statistical framework to remove reporting delays. After validating our framework on synthetic data, we move on to remove and analyze reporting delays in eight hotspots of the COVID-19 pandemic. We conclude our work with a discussion of the impact of delay removal on the accuracy of epidemic forecasts.

## Results

### Notation

Before presenting our findings, we introduce our notation for the epidemic data. Throughout the text, we operate with the infectious I, recovered R, and deceased D data. Each dataset is a time series of values, each corresponding to a specific observation time. For brevity, we refer to the triplet of infectious, recovered, and deceased data as Y={I,R,D}. All values contained in the *Y* time series are fractions of individuals found in the corresponding state on a specific day. For instance, I[k] corresponds to the fraction of individuals who are infectious on day *k*. Since COVID-19 reported data often is advertised in the form of changes in the number of epidemic cases, we find it convenient to introduce the daily changes in epidemic data as ΔY[k] for Y={I,R,D}. Further, in this work, we operate with reported epidemic data $\tilde{Y}$ and inferred data $\hat{Y}$. Since reported and inferred data are expected to differ from the true data *Y*, we need to distinguish the three. We summarize our notation in Table 1.

**Table 1.** Naming convention for the epidemic data, Y={I,R,D}.

Cumulative quantities	Quantity increments
*Y*: fractions of cases	ΔY : fractions of new cases
$\tilde{Y}$ : fractions of reported cases	$\Delta \tilde{Y}$ : fractions of reported new cases
$\hat{Y}$ : fractions of predicted cases	$\Delta \hat{Y}$ : fractions of predicted new cases

### Evidence for reporting delays in epidemiological data

**Fig. 1.**
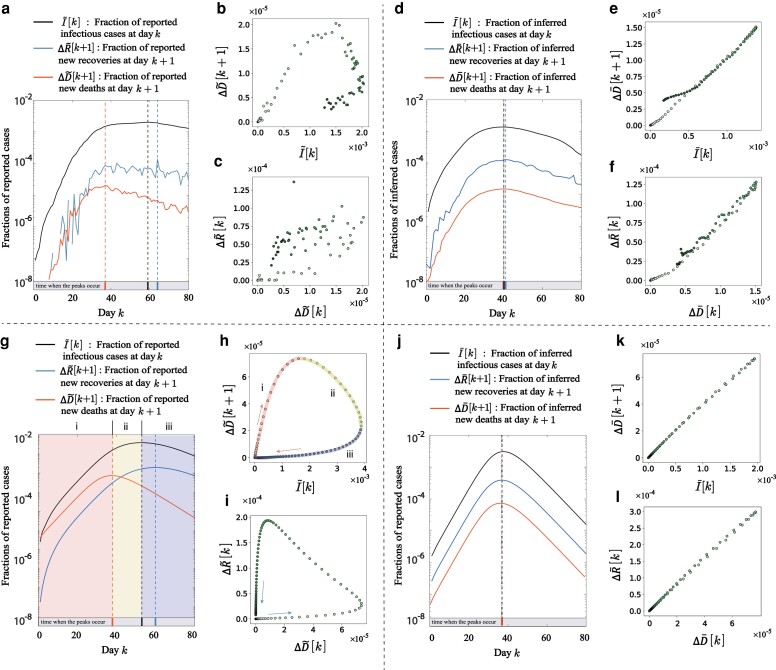
Evidence for reporting delays in COVID-19 epidemic data. a) Reported infectious $\tilde{I}[k]$, recovered $\Delta \tilde{R}[k+1]$ and deceased $\Delta \tilde{D}[k+1]$ cases for the first COVID-19 wave in Spain. The k=0 day corresponds to 2020 February 25. Note the difference in peaks of the reported data. b) and c) display pairwise color-coded scatter plots of $\Delta \tilde{D}[k+1]$ vs. $\tilde{I}[k]$, and $\Delta \tilde{R}[k]$ vs. $\Delta \tilde{D}[k]$ for Spain. Colors, from light to dark green, reflect different days in the data ranging, respectively, from k=0 to k=80. The scatter plots in b) and c) form, respectively, clockwise and counterclockwise loop patterns. d)–f) display the epidemic data in Spain after the removal of reporting delays. g) Synthetic epidemic data generated with the SIRD model by solving Eq. 1 with parameters β=0.5, $\gamma_{r}=0.2$, $\gamma_{d}=0.05$, R[0]=D[0]=0, $I[0]=10^{-6}$, and S[0]=1−I[0]. Synthetic reporting delays were generated with the Pólya-Aeppli distributions using Eq. 3 with parameters $\lambda_{I}=3.68$, $\theta_{I}=0.5$, $E[T_{I}]=7.36$; $\lambda_{R}=9.46$, $\theta_{R}=0.4$, $E[T_{R}]=23.65$; $\lambda_{D}=0.3$, and $\theta_{D}=0.3$, $E[T_{D}]=1$. h) and i) display pairwise color-coded scatter plots of $\Delta \tilde{D}[k+1]$ vs. $\tilde{I}[k]$, and $\Delta \tilde{R}[k]$ vs. $\Delta \tilde{D}[k]$ for the SIRD epidemic data. j)–l) display the synthetic epidemic data after the removal of reporting delays.

We begin the exposition by considering daily reports on the fractions of infected $\Delta \tilde{I}$, recovered $\Delta \tilde{R}$, and deceased $\Delta \tilde{D}$ individuals in Spain. We used the daily epidemic reports in Spain to construct the fraction of infected individuals as $\tilde{I}[k]=\sum_{\ell =0}^{k-1}(\Delta \tilde{I}[\ell ]-\Delta \tilde{R}[\ell ]-\Delta \tilde{D}[\ell ])$. While both the infected $\tilde{I}$ and the deceased data $\tilde{D}$ indicate that the first COVID-19 wave in Spain peaked in April 2020, the exact timings of the two peaks are, nevertheless, different. As observed in Fig. 1a, reported new deceased cases $\Delta \tilde{D}$ reached their peak on 2020 April 1st, while the highest fraction of infectious individuals $\tilde{I}$ was observed 22 days later on 2020 April 23. This observation is not specific to Spain: the peaks in the number of reported new deceased cases $\Delta \tilde{D}$ precede those of infectious cases by more than 1 week in most regions, Fig. S1. We make similar observations for COVID-19 daily recovery reports $\Delta \tilde{R}$, which exhibit their peaks after those of the deceased cases $\Delta \tilde{D}$, Figs. 1a and S1. Furthermore, when plotted as a function of daily deceased cases $\Delta \tilde{D}$, infectious cases $\tilde{I}$ and daily recovered cases $\Delta \tilde{R}$ form “loop” patterns, see Figs. 1b, c and S2.

The patterns observed in Figs. 1a–c, S1, and S2 may indicate the presence of reporting delays. Indeed, epidemic models view recoveries and deaths of patients as stochastic processes with effective rates proportional to the number of infected individuals *I*, $\Delta R[k]\propto \gamma_{r}I[k]$, $\Delta D[k]\propto \gamma_{d}I[k]$. Since the SARS-CoV-2 virus has hardly changed during the first wave of the pandemic, we expect that recovery $\gamma_{r}$ and death $\gamma_{d}$ rates are approximately constant during this period (40–42). This observation suggests that changes in the fractions of recovered ΔR, and deceased ΔD data are proportional to the fraction of infectious individuals I and, therefore, reach maximum values at the same time step, contradicting Fig. 1a–c.

We hypothesize that the disagreement between $\Delta \tilde{R}$, $\Delta \tilde{D}$, and $\tilde{I}$ is due to reporting delays. If each $\Delta \tilde{R}$, $\Delta \tilde{D}$, and $\tilde{I}$ time series are reported with different delays, their peaks are expected to differ. Likewise, the loop patterns of Fig. 1b, c may also be the result of an effective time shift between two nonmonotonous time series.

To check if reporting delays may result in the observed patterns, we consider the compartmental Susceptible-Infectious-Recovered-Deceased (SIRD) epidemic model. Within the SIRD model, the population is split into four compartments: susceptible *S*, infectious *I*, recovered *R*, and deceased *D*. Compartment *S* denotes the fraction of susceptible individuals, who can be infected by infectious individuals. Compartment *I* denotes the fraction of individuals, who have been infected but have not recovered or are deceased. Compartments *R* and *D* are respectively the fractions of individuals, who have recovered or are deceased. The SIRD model assumes that recovered individuals become immune and cannot be infected by the virus in the future. Further, the SIRD model assumes the uniform mixing of the Infectious and Susceptible sub-populations. As a result, the discrete-time transitions between the compartments are governed by first-order difference time equations


(1) $$\begin{array}{rl} & I[k+1]-I[k]=\beta I[k]S[k]-(\gamma_{r}+\gamma_{d})I[k],\\ & R[k+1]-R[k]=\gamma_{r}I[k],\\ & D[k+1]-D[k]=\gamma_{d}I[k],\\ & S[k]+I[k]+R[k]+D[k]=1, \end{array}$$

where *β*, $\gamma_{r}$, and $\gamma_{d}$ are the infection, the recovery, and the deceased probabilities, respectively. We used Eq. 1 to generate infectious I[k], recovered R[k], and deceased D[k] time series epidemic data, and then added synthetic delays to the time series data, see Fig. 1g–i and Supplementary Material B. Since reporting delays were manually added, we observe that ΔR[k+1], ΔD[k+1], and I[k] reach maxima at different time steps, Fig. 1g.

Upholding our hypothesis, we observe the formation of loop patterns in the $\Delta \tilde{D}[k+1]$ vs. $\tilde{I}[k]$ and $\Delta \tilde{R}[k+1]$ vs. $\tilde{D}[k]$ in Fig. 1h, i, similar to those of Spain, Fig. 1b, c. The observed loop patterns are due to the effective horizontal shifts of the corresponding times series due to reporting delays. Indeed, let us split the observation time window into three windows formed by the maxima of the $\Delta \tilde{D}[k+1]$ and $\tilde{I}[k]$ curves, as shown in Fig. 1g. In window *i*, both $\tilde{I}[k]$ and $\Delta \tilde{D}[k+1]$ increase as a function of discrete time *k*. Since reporting delays in the synthetic $\Delta \tilde{D}[k+1]$ data are smaller than those in $\tilde{I}[k]$, this time window corresponds to the upper branch of the $\Delta \tilde{D}[k+1]$ vs. $\tilde{I}[k]$ loop in Fig. 1h. In window *ii*, $\tilde{I}[k]$ increases while $\Delta \tilde{D}[k+1]$ decreases. Thus, window *ii* corresponds to the top (decreasing) section of the $\Delta \tilde{D}[k+1]$ vs. $\tilde{I}[k]$ loop, Fig. 1h. Finally, in window *iii* both $\Delta \tilde{D}[k+1]$ and $\tilde{I}[k]$ decrease as a function of time step *k* resulting in the lowest section of the $\Delta \tilde{D}[k+1]$ vs. $\tilde{I}[k]$ loop, Fig. 1h. Combined, all sections correspond to the loop pattern of Fig. 1h with points progressing in the clockwise direction. Similar considerations explain the counterclockwise loop pattern in the $\Delta \tilde{R}[k+1]$ vs. $\tilde{D}[k]$ scatter plot. The counterclockwise progression of points in this loop pattern is due to $\Delta \tilde{R}[k]$ lagging behind the $\Delta \tilde{D}[k]$ time series, Fig. 1i.

### A null model for reporting delays

**Fig. 2.**
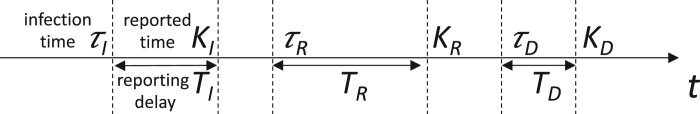
A schematic representation of epidemic events and corresponding delay times.

To uncover reporting delays, we employ the following null model: each individual *i* is endowed with random event time $\tau_{Y_{i}}$ of either getting infected, $\tau_{I_{i}}$, recovered, $\tau_{R_{i}}$, or deceased, $\tau_{D_{i}}$. Figure 2 depicts the random delay time for event $Y_{i}=\{I_{i},R_{i},D_{i}\}$ as $T_{Y_{i}}=\{T_{I_{i}},T_{R_{i}},T_{D_{i}}\}$ and the corresponding random reported time as $K_{Y_{i}}=\tau_{Y_{i}}+T_{Y_{i}}$. Within our discrete-time setting, these random times are discrete random variables. Assuming that the reporting delays $T_{Y_{i}}$ are independent of the events $Y_{i}$, we obtain for the reported time $K_{Y_{i}}$


(2) $$\mathrm{Pr}\left[K_{Y_{i}}=k\right]=\sum_{y=0}^{\infty }\sum_{m=0}^{\infty }\mathrm{Pr}\left[\tau_{Y_{i}}=y\right]\mathrm{Pr}\left[T_{Y_{i}}=m\right]\delta_{y+m,k}=\sum_{m=0}^{k}\mathrm{Pr}\left[\tau_{Y_{i}}=k-m\right]\mathrm{Pr}\left[T_{Y}=m\right].$$

By taking the arithmetic mean of Eq. 2, we find that the expected reported fraction $\Delta \tilde{Y}[k]=\frac{1}{N}\sum_{i}\mathrm{Pr}[K_{Y_{i}}=k]$ of individuals in state *Y* is a discrete convolution


(3) $$\Delta \tilde{Y}[k]=\sum_{m=0}^{k}\mathrm{Pr}\left[T_{Y}=m\right]\Delta Y[k-m],$$

where Y={I,R,D}. Equation 3 serves as the foundation for uncovering reporting delays and improving epidemic forecasts.

### Statistical framework to uncover reporting delays

The delay distribution $\mathrm{Pr}[T_{Y}=m]$ in (3) can be determined, in principle, as the solution of a large set of quadratic equations, deduced from the governing epidemic equations, as shown in Supplementary Material D, provided that we assume a same distribution for each delay $T_{Y_{i}}=\{T_{I_{i}},T_{R_{i}},T_{D_{i}}\}$. Since that solution is rather demanding, we propose a simplification. We assume that the delay distribution $\mathrm{Pr}[T_{Y}=m]$ is generated by a two-parameter probability distribution with parameters κ={λ,θ}, which is sufficiently general to incorporate the real probability distribution of the delay.

We consider three families of two-parameter discrete probability distributions: the negative binomial distribution, the Neyman type A distribution, and the Pólya-Aeppli distribution, see Materials and methods A. These distributions are quite general and contain some well-known 1-parameter distributions as special cases. The logarithmic, geometric, and Poisson distributions are, for instance, all special cases of the negative binomial distribution (43). The three distributions are sufficiently general to generate data with variable mean and variance and of varying skewness (43, 44)—properties expected of the epidemic delay data, see Materials and methods A. For brevity, we focus on the Pólya-Aeppli distribution in the main text and report the results for the other two distributions in Fig. S4.

In our statistical framework, we assume that the reporting delays correspond to three datasets, Y={I,R,D}, which are all characterized by the Pólya-Aeppli distribution, albeit with different parameters $\kappa_{Y}\equiv \{\lambda_{Y},\theta_{Y}\}$. Hence, there are in total six parameters for three Pólya-Aeppli distributions, $\kappa =(\lambda_{I},\theta_{I},\lambda_{R},\theta_{R},\lambda_{D},\theta_{D})$. With the choice of the Pólya-Aeppli distribution, the reporting delays $\Delta \tilde{Y}$ in our basic equation Eq. 3 can be determined in the parameterized form $\Delta {\tilde{Y}}_{\kappa }$.

Given the incremental time series $\Delta {\tilde{Y}}_{\kappa }$, the cumulative time series ${\tilde{Y}}_{\kappa }$ are given by


(4) $$\begin{array}{rl} & {\tilde{I}}_{\kappa }[k+1]={\tilde{I}}_{\kappa }[k]+\Delta {\tilde{I}}_{\kappa }[k]-\Delta {\tilde{R}}_{\kappa }[k]-\Delta {\tilde{D}}_{\kappa }[k],\\ & {\tilde{R}}_{\kappa }[k+1]={\tilde{R}}_{\kappa }[k]+\Delta {\tilde{R}}_{\kappa }[k],\\ & {\tilde{D}}_{\kappa }[k+1]={\tilde{D}}_{\kappa }[k]+\Delta {\tilde{D}}_{\kappa }[k], \end{array}$$

for k≥0, and $\tilde{I_{\kappa }}[0]=\tilde{R_{\kappa }}[0]=\tilde{D_{\kappa }}[0]=0$.

The main assumption of our reporting delay removal framework is that the increments in new recovered ΔR and deceased ΔD individuals are proportional to the cumulative fraction of infectious individuals *I*.

Therefore, we determine the “best” parameters $\bar{\kappa }$ that maximize the product of pairwise correlations among the three epidemic time series in $\tilde{Y_{\kappa }}$:


(5) $$O_{b}(\tilde{Y_{\kappa }})\equiv O_{b}(\tilde{I_{\kappa }},\Delta \tilde{R_{\kappa }},\Delta \tilde{D_{\kappa }})=\rho (\Delta \tilde{R_{\kappa }},\Delta \tilde{D_{\kappa }})\rho (\tilde{I_{\kappa }},\Delta \tilde{R_{\kappa }})\rho (\tilde{I_{\kappa }},\Delta \tilde{D_{\kappa }}),$$

where ρ(X,Y) is the Pearson correlation coefficient between time series *X* and *Y*. The objective function $O_{b}(\tilde{Y_{\kappa }})$ in Eq. 5 reaches its maximum value of 1 when all three pairwise correlations among the $\tilde{I_{\kappa }}$, $\Delta \tilde{R_{\kappa }}$ and $\Delta \tilde{D_{\kappa }}$ time series are 1, which we expect when recovery $\gamma_{r}$ and deceased $\gamma_{d}$ epidemic probabilities are constant. Due to the nature of the Pearson correlation coefficient, the objective function $O_{b}(\tilde{Y_{\kappa }}[k])=O_{b}(\tilde{Y_{\kappa }}[k-T])$ is invariant under the constant time shift *T* of the epidemic data. As a result, we can only infer the reporting delays up to a constant time shift *T*.

To maximize $O_{b}(\tilde{Y_{\kappa }})$, the random search (45) method is applied: we conduct a large set of independent random iterations, ℓ=1,…,L. At each iteration ℓ, we select the elements of the parameter vector $\bar{\kappa }$ uniformly at random from the prescribed domain of values. Further, at each iteration ℓ, we use the selected parameter set ${\bar{\kappa }}_{\ell }$ to reconstruct the original epidemic data $\Delta {\tilde{Y}}_{{\bar{\kappa }}_{\ell }}$ by solving Eq. 3 and convert incremental data $\Delta {\tilde{Y}}_{{\bar{\kappa }}_{\ell }}$ to cumulative data ${\tilde{Y}}_{{\bar{\kappa }}_{\ell }}$ by solving Eq. 4. We then compute the pairwise correlations in ${\tilde{Y}}_{{\bar{\kappa }}_{\ell }}$ to obtain the objective function $O_{b}({\tilde{Y}}_{{\bar{\kappa }}_{\ell }})$. After completing all random search iterations, the resulting delay parameter $\hat{\kappa }$ is the one maximizing the objective function, $\hat{\kappa }={\mathrm{argmax}}_{{\bar{\kappa }}_{\ell }}O_{b}({\tilde{Y}}_{{\bar{\kappa }}_{\ell }})$, see Materials and methods B.

### Tests on synthetic data

Before uncovering reporting delays in real epidemic data, we test our framework on synthetic datasets, which we generate with the SIRD compartmental model, Eq. 1.

**Fig. 3.**
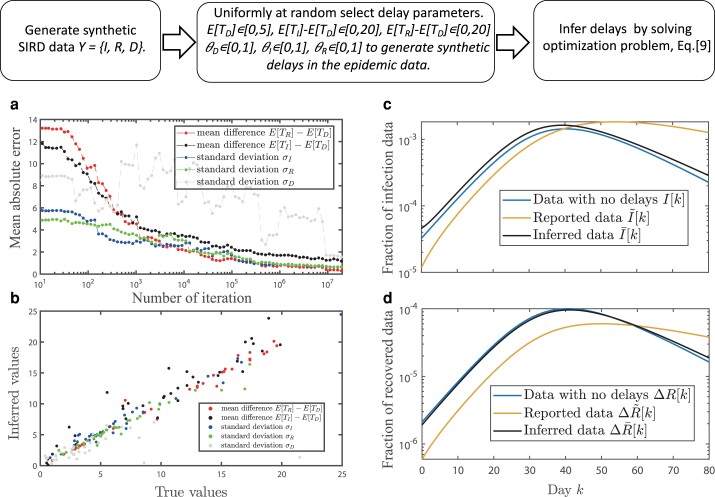
Uncovering reporting delays in synthetic data. We generate epidemic data using the SIRD model, Eq. 1 with parameters β=0.23, $\gamma_{r}=0.079$, and $\gamma_{d}=0.11$ and variable delay parameters. The schematics on top illustrates the process of generation and inference on synthetic data. a) The mean absolute inference errors as functions of the number of iteration steps in the random search. Due to the nature of the Pearson correlation coefficient, it is only possible to infer relative delays, $E[T_{R}]-E[T_{D}]$, $E[T_{I}]-E[T_{D}]$, and $E[T_{R}]-E[T_{I}]$. b) The relationship between the true and the inferred values of delay parameters. c, d) An example of an SIRD synthetic data c) before and d) after the removal of synthetic reporting delays.

To test our framework, we generated 50 SIRD epidemic model datasets with different epidemic parameters and added synthetic reported delays to the obtained times series, as prescribed by Eq. 3. The resulting delay-perturbed synthetic data is fed to our statistical framework that infers the reported synthetically added delays. Figure 1d–f and j–l display, respectively, the reconstructed real and synthetic epidemic data. We find strong correlations between the reconstructed $\Delta \tilde{R}$, $\Delta \tilde{D}$, and $\tilde{I}$ data. Further, Fig. 3a, b indicate that the inferred delay parameters are in good agreement with the true parameters that generated the datasets. Figure 3 illustrates that the inference errors decrease fast as a function of the number of iteration steps saturating at the value of 2 days after $10^{7}$ iterations. To assess the robustness of the inference procedure, we have conducted cross-inference experiments by generating synthetic data with one delay distribution and inferring delays using another distribution, arriving at similar results in Fig. S4.

### Uncovering reporting delays in real data

After testing our inference framework on synthetic data, we moved on to uncover reporting delays in real epidemic data that we have collected from eight regions worldwide, Supplementary Material A. Figure 1d–f and j–l display the reconstructed epidemic data for Spain and for synthetic SIRD model after the identification and removal of reporting delays, see Table S1 for the inferred parameters. Consistent with our expectations, the reconstructed time series of infected I[k], new recovered ΔR[k+1], and new deceased ΔD[k+1] individuals are strongly correlated, Fig. 1e, f and k, l, and their peaks co-occur, as seen in Fig. 1d, j.

We summarize the properties of uncovered reporting delays in the eight regions in Table 2. We find that the infectious and recovered data delays are longer than those for deceased cases and vary from several days to several weeks. This observation is hardly surprising. Indeed, there are several factors contributing to the delays of infectious cases. One factor is the delay between an individual becoming infectious, and the symptom onset (46). Another factor, particularly significant in the first COVID-19 wave, is the delay between the symptom onset and the test result (47). In their turn, delays in the recovered events are likely caused by hospital discharge policies. Indeed, the recovered data are usually derived from hospital discharge events, which occur after the patient’s recovery.

**Table 2.** Inferred reporting delays.

Regions	$E[T_{R}]-E[T_{D}]$	$E[T_{I}]-E[T_{D}]$	$\sigma_{I}$	$\sigma_{R}$	$\sigma_{D}$	$\rho (\Delta \bar{R},\Delta \bar{D})$	$\rho (\bar{I},\Delta \bar{R})$	$\rho (\bar{I},\Delta \bar{D})$	$O_{b}(\bar{Y})$
Italy	28.52	6.59	5.08	27.79	0.72	0.91	0.94	0.99	0.84
Spain	22.66	6.36	8.63	28.39	0.73	0.99	0.99	1.00	0.98
Wuhan	14.80	20.64	51.67	16.13	23.87	0.78	0.89	0.91	0.63
Turkey	14.13	24.85	43.89	12.47	11.21	0.91	0.95	0.98	0.85
Hubei	9.96	21.07	78.62	10.89	54.14	0.85	0.90	0.92	0.71
Romania	3.30	19.05	43.08	90.12	0.29	0.81	0.89	0.97	0.70
Germany	2.72	16.55	39.25	106.89	4.31	0.87	0.88	0.99	0.76
Denmark	0.17	25.07	36.90	2.71	28.25	0.83	0.93	0.94	0.72

The table displays the inferred differences between the expected delay times $E[T_{R}]-E[T_{D}]$, $E[T_{I}]-E[T_{D}]$, standard deviations $\sigma_{I}$, $\sigma_{R}$ and $\sigma_{D}$, and optimized values for the objective function $O_{b}(\bar{Y})$. See Tables S1 and S2 for the corresponding parameters of the Pólya-Aeppli distributions.

Based on the expected delay values, one can naturally split the eight ROIs into three categories: (i) large infectious and small recovered delays: Romania, Germany, and Denmark, (ii) small infectious and large recovered delays: Italy and Spain, and (iii) large infectious and recovered delays: Wuhan, Hubei, and Turkey.

Small infectious delays imply that COVID-19 tests are timely and accurate. This seems to be the case for Italy, which executed more tests per capita in April 2020 than other countries (48). On the contrary, the testing ability of the Hubei province was significantly insufficient during the first wave of the COVID-19 outbreak.

Large delays in the recovered data may be attributed to strict hospital discharge policies. Discharge policy in Italy, for instance, was based on the negative test (49), and it has been shown that COVID-19 tests may stay positive for an extended time after COVID-19 symptoms disappear. In contrast, discharge policies in Denmark were based not on the negative test but on patient symptoms (49), likely leading to shorter reporting delays in the recovered data.

Large standard deviations in the reporting delays may indicate irregularities in reporting mechanisms. As an example, Hubei province expanded its daily testing capacity from 200 to 2,000 individuals from the beginning of the pandemic until January 27th (50). As a result, fewer individuals were tested late at the end of the first COVID-19 wave compared to its beginning, likely resulting in the large standard deviation of infectious data delays.

The small standard deviation in the delays of recovered data observed in Hubei and its capital Wuhan is likely the consequence of the strict discharge criteria (51). Although strict discharge policies cause significant delays, these delays are similar, resulting in relatively smaller $\sigma_{R}$ values. In contrast to Chinese regions, the recovery data for Germany are not reported directly but instead are estimated by a not explained algorithm (52), resulting in large errors and, consequently, larger $\sigma_{R}$ values. Based on the optimized values $O_{b}(\bar{Y})$ as shown in Table S2, the optimization performances of our algorithm for Italy, Spain, and Turkey are better than the other countries.

### Improving epidemic forecasts

Is accounting for reporting delays likely to improve epidemic forecasts? To answer this question, we designed two experiments. Experiment 1 aims to forecast the epidemics ignoring reporting delays and serves as a baseline for Experiment 2, which uncovers reporting delays prior to forecasting epidemic data.

**Fig. 4.**
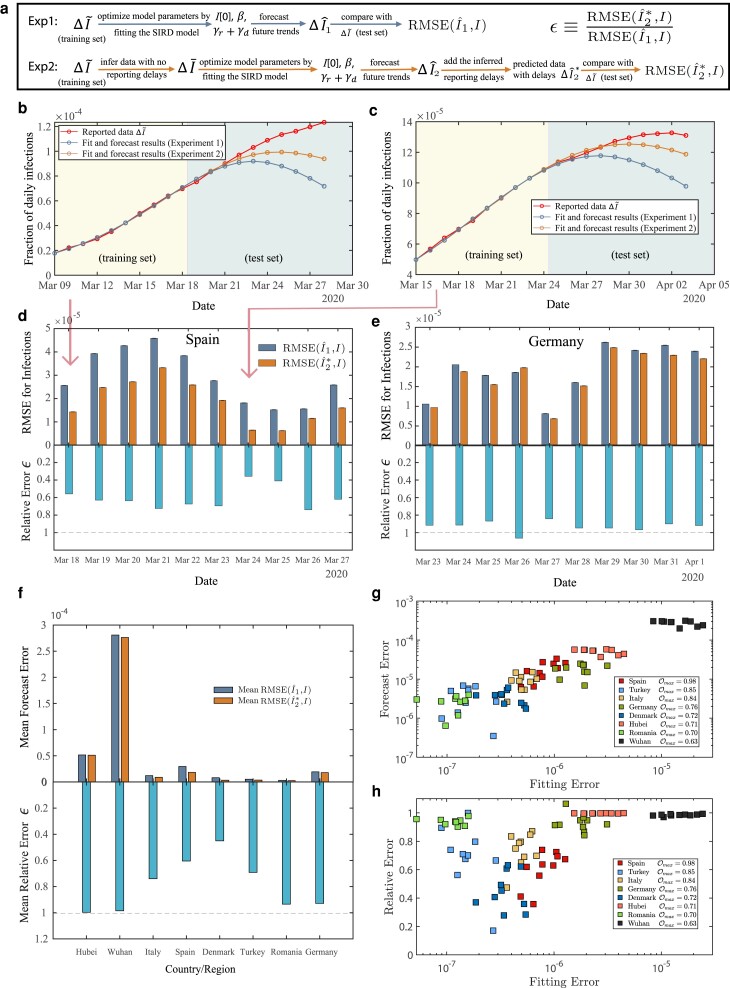
Accounting for reporting delays improves epidemic forecasts. a) The schematic diagram for the two forecast experiments. b), c) display the results of epidemic forecasts in Spain with different forecast start dates. The forecasts of experiment 2 (yellow) are closer to the reported data (red) than the forecasts of experiment 1 (blue). The forecast results for all eight countries or regions are shown in Figs. S5 and S6. d), e) All forecast results for Spain and Germany. Shown are the RMSE forecast errors and their ratios. h) Average forecast errors ϵ for the 8 regions. The benefits of removing reporting delays vary by region. g) The average RMSE forecast error of experiment 2 as a function of SIRD model fitting error for all experiments. h) The mean relative forecast error as a function of SIRD model fitting error for all experiments.

In both experiments, we split the reported epidemic data into two parts, which we call the training and the testing sets, respectively, see Fig. 4a. In Experiment 1, we fit the training set with the SIRD epidemic model, obtaining model parameters *β*, $\gamma_{r}+\gamma_{d}$, and the fraction of initial infected cases I[0]. We then use the SIRD model with the obtained parameters to forecast the epidemic data, Supplementary Material C.

In Experiment 2, we first use the reported data $\tilde{Y}$ in the training set to infer the parameters of the delay distributions $\bar{\kappa }$. We rely on these parameters to remove reporting delays from the testing set and obtain reconstructed data $\Delta \bar{I}$. In the next step, we fit the reconstructed data $\Delta \bar{I}$ with the SIRD model, obtaining *β*, $\gamma_{r}+\gamma_{d}$ and I[0] spreading parameters. We use these spreading parameters to forecast the epidemic data $\Delta \hat{I}$, which we compare to the reported data $\Delta \tilde{I}$ in the testing set. Since $\Delta \tilde{I}$ in the testing set contains the reporting delays, while the forecast $\Delta \hat{I}$ does not, we added the inferred reporting delays back to the $\Delta \hat{I}$ using Eq. 3, obtaining $\Delta {\hat{I}}^{*}$, See Fig. 4a and Supplementary Material C.

Figure 4b, c presents the results of the forecast experiments for Spain, indicating that correcting for reporting delays does improve the forecast accuracy. To evaluate the forecast errors in a systematic way, we evaluated the root mean square errors between the forecasts $I_{F}$ and the testing $I_{T}$ sets:


(6) $$\mathrm{RMSE}(I_{F},I_{T})={\left[\frac{1}{n}\sum_{k=0}^{n-1}(I_{F}[k]-I_{T}[k]{)}^{2}\right]}^{\frac{1}{2}},$$

where *n* is the size of the testing set. Further, to quantify the benefits of accounting for reporting delays, we used the ratio of the root mean square errors measured in forecasts with and without reporting delays, $\epsilon \equiv \frac{\mathrm{RMSE}({\hat{I}}_{2}^{*},I)}{\mathrm{RMSE}({\hat{I}}_{1},I)}$, where ${\hat{I}}_{1}$ and ${\hat{I}}_{2}^{*}$ are the epidemic forecasts obtained in experiments 1 and 2, respectively. The smaller the ratio, the smaller the relative forecast error.

We measured the accuracy of epidemic forecasts with different start dates for Spain in Germany. While we did observe a substantial improvement in forecast accuracy for Spain, Fig. 4d, this was not the case for Germany, where the removal of reporting delays only improved the forecasts by a small margin, Fig. 4e. On a broader scale, we observed that the benefits of correction for reporting delays vary across all regions of interest, Figs. 4f, S5, and S6. While there is nearly a two-fold improvement in the forecast accuracy for Denmark, there is little improvement for Wuhan and Hubei.

Apart from only measuring and testing a small part of the population—which might be too small or not sampled adequately—there are at least two factors that may hinder epidemic forecast accuracy. The first factor is the insufficient accuracy of reporting delay removals. The second one is the inability of the SIRD model to reproduce the COVID-19 dynamics accurately. We can quantify the former by the maximum attained value of the objective function $O_{b}(\bar{Y}{)}_{\max}$ that we aim to maximize when removing reporting delays. While there is no direct way to quantify the goodness of the SIRD model in epidemic forecasts, as an indirect measure, we fit the training set with the SIRD model and compute the fitting error.

Figure 4g shows that the forecast error is strongly correlated with the SIRD fitting error, Pearson r=0.89, $p<10^{-27}$, indicating that the highest epidemic forecast accuracy is attained when the model fit is accurate. The forecast error ratio depends both on the $O_{b}(\bar{Y}{)}_{\max}$ and SIRD model fitting, Fig. 4h. We observe the largest error ratio ϵ in Wuhan, Hubei, and Romania, Fig. 4h. Wuhan and Hubei correspond to the largest SIRD fitting errors, while Romania corresponds to the lowest SIRD fitting errors. At the same time, all three regions are quantified by the lowest $O_{b}(\bar{Y}{)}_{\max}$ values. The other five regions, corresponding to smaller error ratios, are characterized by significantly larger $O_{b}(\bar{Y}{)}_{\max}$ values, Fig. 4g and Table S2.

These observations indicate that $O_{b}(\bar{Y}{)}_{\max}$ may be used as an early indicator of the benefit of delay removals. Indeed, lower $O_{b}(\bar{Y}{)}_{\max}$ values may indicate that reporting delays were not removed successfully or the initial assumption of the proportionality among *I*, ΔR, and ΔD times series does not hold. We note that high $O_{b}(\bar{Y}{)}_{\max}$ values correspond to high benefits of delay removals regardless of the SIRD fitting error. In the case of Romania, for instance, SIRD fitting errors are among the smallest, resulting in low forecast errors. Yet, the removal of reporting delays does not result in even lower forecast errors.

There might be multiple reasons for suboptimal reporting delay removals in the case of Hubei, Wuhan, and Romania. One possibility is the nonstationary nature of delays. The main assumption of our framework is that delay times are independent and drawn from the same distribution. While COVID-19 did not significantly mutate over the short time span of the first wave, our ability to handle the infections improved significantly. The PCR test capacity in China has grown remarkably during the first COVID wave, possibly explaining the limited effect of reporting delays on improving epidemic forecasts in Hubei and Wuhan.

### Discussion

Data delays are ubiquitous in data sciences and adversely affect data analysis (53). The unique property of the epidemic data, enabling us to identify and filter out reporting delays, is the proportionality between the number of infectious individuals *I* and the rates of change in the deceased ΔD and the recovered ΔR individuals.

We relied on this proportionality property to develop a parametric statistical framework to uncover reporting delays in the first COVID-19 wave and applied it to eight regions significantly affected during the first COVID-19 wave. The character of uncovered delays varies across studied regions and can be explained by region-specific medical capacities and epidemic policies.

Concerning the epidemic forecasts, we found that the benefits of using curated epidemic data, as opposed to the raw data, are maximized in situations when reporting delays are removed efficiently. One of the main factors hindering the efficiency of delay removal—the nonstationarity of reporting delays—can be caused by either varying spreading properties of the virus or by rapid changes in medical capacities or epidemic restrictions across the regions. While the spreading properties of COVID-19 did not change significantly during the first wave of the pandemic, both medical capacities and epidemic policies were the subjects of constant updates as the communities learned how to handle the pandemic.

We expect that our framework will prove most useful in the early stages of an epidemic when accurate epidemic forecasts are essential to plan intervention strategies and raise public awareness. Common sense dictates that data collection and reporting challenges are the most significant during that time.

Our statistical framework is based on the assumption that transition probabilities $\gamma_{r}$ and $\gamma_{d}$ quantifying transitions from the infectious to the recovered, I→R and the infections to the deceased, I→D states, respectively are constant across the population and do not change over the observational period. While these assumptions are approximations, we expect them to hold better in early epidemic stages than in later stages. In the later stages, the population becomes more heterogeneous, e.g. due to natural or vaccine-induced immunity (54). In these situations, our framework can be generalized by splitting the infectious compartment *I* into sub-compartments and considering different rates, e.g.


(7) $$\begin{array}{rl} & \Delta R[k]=\gamma_{1,r}I_{1}[k]+\gamma_{2,r}I_{2}[k],\\ & \Delta D[k]=\gamma_{1,d}I_{1}[k]+\gamma_{2,d}I_{2}[k],\\ & I[k]=I_{1}[k]+I_{2}[k], \end{array}$$

where $I_{1}[k]$ and $I_{2}[k]$ are two infectious sub-compartments characterized by different recovery and death probabilities.

At the same time, we stress that our statistical framework does not make strong assumptions about the spreading mechanisms of a pathogen and can be used in combination with any forecasting method that requires infectious, recovered, and deceased data as input.

In conclusion, our framework can be used as a preliminary filter for any epidemic forecast tool that takes infected, recovered, and deceased data as input. Our framework holds for any compartmental epidemic model, provided that each compartment can be measured, because a time series of each compartment is indispensable. Accurate and timely epidemic forecasts are of immense value for society and policymakers to minimize the adverse effects of the virus.

## Materials and methods

### Types of distributions

To determine the family of reporting delay distributions that best suit our data, we consider three different two-parameter discrete distributions (55) below:

(I) Negative binomial distribution.


(8) Pr[T=m]=(m+r−1m)(1−p)mpr.

The negative binomial distribution with parameters r>0 and p∈[0,1] has mean value E[T]=r(1−p)/p and variance $\mathrm{Var}[T]=r(1-p)/p^{2}$.

(II) Pólya-Aeppli distribution is also known as the geometric Poisson distribution.


(9) Pr[T=m]={∑j=1me−λλjj!(1−θ)m−jθj(m−1j−1),m>0e−λ,m=0.

The Pólya-Aeppli distribution with parameters λ>0 and θ∈[0,1] has mean value E[T]=λ/θ and variance $\mathrm{Var}[T]=\lambda (2-\theta )/\theta^{2}$.

(III) Neyman type A distribution.


(10) $$\mathrm{Pr}[T=m]=\frac{\mu^{m}e^{-\xi }}{m!}\sum_{\;j=0}^{\infty }\frac{{\left(\xi e^{-\mu }\right)}^{j}}{j!}j^{m}.$$

The Neyman type A distribution with parameters ξ>0 and μ>0 has mean value E[T]=ξμ and variance Var[T]=ξμ(1+μ).

### Inferring reporting delays

To uncover reporting delays in epidemic data, we determine parameters of delays distributions $\kappa_{Y}$ maximizing the pairwise correlations between the epidemic time series, Eq. 5. This optimization problem can be compactly written as:


(11) $$\begin{array}{rl} \underset{\kappa }{\text{arg max}} & \;O_{b}(Y)\equiv \rho (\Delta R,\Delta D)\rho (I,\Delta R)\rho (I,\Delta D)\\ \text{s.t.} & \;\Delta I=\Psi_{I}^{-1}\Delta \tilde{I},\Delta R=\Psi_{R}^{-1}\Delta \tilde{R},\Delta D=\Psi_{D}^{-1}\Delta \tilde{D},\\ & \;\min(\Delta I[k],\Delta R[k],\Delta D[k],I[k])\geq 0,\;\text{for}\;k=1,\ldots ,T. \end{array}$$

Here *T* is the size of epidemic time series, $\tilde{Y}\equiv \{\tilde{I},\tilde{R},\tilde{D}\}$ and Y≡{I,R,D} are reported and reconstructed epidemic data, respectively, while $\Delta Y=\Psi_{Y}^{-1}\Delta \tilde{Y}$ are the matrix solutions of Eq. 3. Indeed, Eq. 3 can be written in the matrix form as $\Delta \tilde{Y}=\Psi_{Y}\Delta Y$ for Y={I,R,D}, where


$$\begin{array}{rl} & {\Psi_{Y}}_{i,j}≜\left\{ \begin{array}{cc} \mathrm{Pr}[T_{Y}=i-j] & \mathrm{if}\;i\geq j.\\ 0 & \mathrm{otherwise}. \end{array}\right., \end{array}$$

Then, $\Psi^{-1}$ is the inverse of $\Psi_{Y}$ and $\Delta Y=\Psi_{Y}^{-1}\Delta \tilde{Y}$.

In the main text, we assume that reporting delays are iid random variables drawn from three Pólya-Aeppli distributions, Eq. 9, with distinct parameters $\left\{\lambda_{I},\theta_{I}\right\}$, $\left\{\lambda_{R},\theta_{R}\right\}$, and $\left\{\lambda_{D},\theta_{D}\right\}$. In the case of Pólya-Aeppli distributions, the parameter vector takes the form of $\kappa \equiv \{\lambda_{I},\theta_{I},\lambda_{R},\theta_{R},\lambda_{D},\theta_{D}\}$. We solve the optimization problem given by Eq. 11 and the random search (45). For each iteration ℓ=1,…,L, we treat the expected values of the Pólya-Aeppli distributions, $E[T_{Y}]=\lambda_{Y}/\theta_{Y}$, as iid random variables and draw from the uniform pdfs U[0,30] for Y={I,R,D}. Similarly, we draw $\theta_{Y}$ parameters independently at random from uniform pdfs U[0,1] and then determine $\lambda_{Y}$ parameters as $\lambda_{Y}=\theta_{Y}E[T_{Y}]$, obtaining $\kappa_{\ell }$. In our experiments, we set the maximum number of search iterations to $L=10^{7}$.

For each $\kappa_{\ell }$, we use Eq. 3 to reconstruct original data $Y_{\ell }$, which we then use to compute the objective function $O_{b}(Y_{\ell })$. After completing all iterations, the thought parameter vector $\hat{\kappa }$ describing reporting delays is the one corresponding to the maximum $O_{b}(Y_{\ell })$ value, $\hat{\kappa }={\text{arg max}}_{\kappa }\;O_{b}(Y)$.

## Supplementary Material

pgae204_Supplementary_Data

## Data Availability

All epidemic data used in this work are publicly available from the original sources, see Supplementary Information A. The extracted epidemic time series for the eight regions of interest are deposited in FigShare (https://doi.org/10.6084/m9.figshare.22639519.v1). Code to generate SIRD epidemic time series and to uncover reporting delays is available on GitHub (https://github.com/qzhszl/Reporting-delays-a-widely-neglected-impact-factor-in-COVID-19-forecasts.git).
