# Supplementary material for: Reporting delays: A widely neglected impact factor in COVID-19 forecasts
Source: PNAS Nexus. 2024 May 22;3(6):pgae204. doi: 10.1093/pnasnexus/pgae204 (PMC11156234; doi:10.1093/pnasnexus/pgae204)
Supplement: pgae204_Supplementary_Data [file pgae204_supplementary_data.pdf]

Supplementary Information for

Reporting delays: a widely neglected impact factor in COVID-19 forecasts

Long Ma, Zhihao Qiu, Piet Van Mieghem, Maksim Kitsak

Maksim Kitsak
E-mail: M.A.Kitsak@tudelft.nl

This PDF file includes:

Supplementary text
Figs. S1 to S6
Tables S1 to S2
SI References

Supporting Information Text

A. Data availability. The data sources of COVID-19 cases for each country/region are as follows:
1. **Italy**: The data was collected daily from Dipartimento della Protezione Civile (http://www.salute.gov.it/portale/home.html) from Feb 21, 2020 to May 4, 2020. The available data can be found in WHO COVID-19 global dataset https://covid19.who.int/WHO-COVID-19-global-data.csv, https://opendatamds.maps.arcgis.com/apps/dashboards/12a643195a994a558f4dbd603338ee33, https://www.worldometers.info/coronavirus/ and https://it.wikipedia.org/wiki/Statistiche_della_pandemia_di_COVID-19_in_Italia
2. **Spain**: The data was collected daily from Ministry of Health (Spain) (https://www.sanidad.gob.es/home.htm) from February 25, 2020 to May 15, 2020. The available data can be found in WHO COVID-19 global dataset https://covid19.who.int/WHO-COVID-19-global-data.csv , https://www.rtve.es/noticias/20230331/mapa-del-coronavirus-espana/2004681.shtml, https://www.worldometers.info/coronavirus/ and https://en.wikipedia.org/wiki/COVID-19_pandemic_in_Spain#Statistics
3. **Wuhan**: The data was collected daily from National Health Commission of the People's Republic of China (http://en.nhc.gov.cn/) from January 18, 2020 to March 15, 2020. The available data can be found in Wuhan Municipal Health Commission https://wjw.wuhan.gov.cn/ztzl_28/fk/yqtb/
4. **Turkey**: The data was collected daily from Ministry of Health (Turkey) (https://www.saglik.gov.tr/) from March 11, 2020 to May 16, 2020. The available data can be found in WHO COVID-19 global dataset https://covid19.who.int/WHO-COVID-19-global-data.csv, https://www.worldometers.info/coronavirus/ and https://en.wikipedia.org/wiki/COVID-19_pandemic_in_Turkey#Statistics
5. **Hubei**: The data was collected daily from National Health Commission of the People's Republic of China (http://en.nhc.gov.cn/) from January 18, 2020 and end on March 15, 2020. The available data can be found in http://www.nhc.gov.cn/yjb/s7860/202205/4b6b9808ba8a469c9026f05d3f35546e.shtml and https://en.wikipedia.org/wiki/Template:COVID-19_pandemic_data/China_medical_cases_by_province
6. **Romania**: The data was collected daily from Ministry of Health (Romania) (http://www.ms.ro/comunicate/) from February 26, 2020 to May 23, 2020. The available data can be found in WHO COVID-19 global dataset https://covid19.who.int/WHO-COVID-19-global-data.csv, https://www.worldometers.info/coronavirus/ and https://en.wikipedia.org/wiki/COVID-19_pandemic_in_Romania#Cumulative_cases
7. **Germany**: The data was collected daily from Robert Koch-Institut (RKI) (https://www.rki.de/DE/Content/InfAZ/N/Neuartiges_Coronavirus/Fallzahlen.html) and new situation reports of the RKI (https://www.rki.de/DE/Content/InfAZ/N/Neuartiges_Coronavirus/Situationsberichte/Gesamt.html). from March 1, 2020 to May 19, 2020. The available data can be found in WHO COVID-19 global dataset https://covid19.who.int/WHO-COVID-19-global-data.csv, https://www.rki.de/DE/Content/InfAZ/N/Neuartiges_Coronavirus/Situationsberichte/Nov_2020/2020-11-11-de.pdf?__blob=publicationFile, https://www.worldometers.info/coronavirus/ and https://en.wikipedia.org/wiki/Statistics_of_the_COVID-19_pandemic_in_Germany.
8. **Denmark**: The data was collected daily from Statens Serum Institut (https://en.ssi.dk/) from February 27, 2020 to May 16, 2020. The available data can be found in WHO COVID-19 global dataset https://covid19.who.int/WHO-COVID-19-global-data.csv, https://covid19.datelazi.ro/, https://www.worldometers.info/coronavirus/ and https://en.wikipedia.org/wiki/COVID-19_pandemic_in_Denmark#Statistics.

We extract the epidemic data time series from these datasets and make them available at https://doi.org/10.6084/m9.figshare.22639519.v1.

B. Qualitative explanation for the formation mechanism of the loop patterns in epidemic data. In the main text, we consider the epidemic data reported for Spain during the first wave of the COVID-19 pandemic, Fig. 1. We attribute the observed misalignment of peaks of the epidemic data as well as the loop patterns to the presence of delays in the reporting data. Our observations are not specific to Spain. As evidenced in Fig. S1 and Fig. S2, we observe similar patterns in all 8 regions of interest.

To verify if the observed patterns could follow from reporting delays, we conduct a test using synthetic epidemic data. In more precise terms, we generated synthetic epidemic time series using the SIRD epidemic model, Eq. [3], setting epidemic parameters to $\beta = 0.5$, $\gamma_r = 0.2$, and $\gamma_d = 0.05$. After obtaining synthetic epidemic times series $Y[k]$, we added reporting delays using Eq. [1]. We assumed that reporting delays follow Pólya-Aeppli distributions with $\zeta_D = 1/3$, $\mu_D = 2$, $\zeta_I = 2$, $\mu_I = 2$, $\zeta_R = 3$, and $\mu_R = 6$, where sub-indices D, I, and R refer to deceased, infected, and recovered sub-populations. These parameters correspond to distributions with $E[T_D] = 2/3$, $\mathrm{Var}[T_D] = 10/9$, $E[T_I] = 4$, $\mathrm{Var}[T_I] = 20$, $E[T_R] = 18$, and $\mathrm{Var}[T_R] = 342$, see Fig. S3(a). Fig. S3(b,c) illustrates that the resulting synthetic data exhibit patterns very similar to those observed for the COVID-19 data.

The formation of the $\Delta\widetilde{R}[k+1]$ versus $\tilde{I}[k]$ and $\Delta\widetilde{D}[k+1]$ versus $\widetilde{I}[k]$ loop patterns in Fig. S3(c) is due to the effective horizontal shifts of the corresponding times series due to reporting delays. Indeed, let us split the observation time window into three windows formed by the maxima of the $\Delta\widetilde{D}[k+1]$ and $\widetilde{I}[k]$ curves, as shown in Fig. S3(b). In window i, both $\widetilde{I}[k]$ and $\Delta\widetilde{D}[k+1]$ increase as a function of discrete time step k. Since reporting delays in the synthetic $\Delta\widetilde{D}[k+1]$ data are smaller than those in $\widetilde{I}[k]$, this time window corresponds to the upper branch of the $\Delta\widetilde{D}[k+1]$ versus $\widetilde{I}[k]$ loop in Fig. S3(c). In window ii, $\widetilde{I}[k]$ increases while $\Delta\widetilde{D}[k+1]$ decreases. Thus, window ii corresponds to the top (decreasing) section of the $\Delta\widetilde{D}[k+1]$ versus $\widetilde{I}[k]$ loop, Fig. S3(c). Finally, in window iii both $\Delta\widetilde{D}[k+1]$ and $\widetilde{I}[k]$ decrease as a function of time step k resulting in the lowest section of the $\Delta\widetilde{D}[k+1]$ versus $\widetilde{I}[k]$ loop, Fig. S3(c). Combined, all sections correspond to the loop pattern of Fig. S3(c). with points progressing in the clockwise direction. Similar considerations explain the counterclockwise loop pattern in the $\Delta\widetilde{R}[k+1]$ vs $\widetilde{I}[k]$ scatter plot. The counterclockwise progression of points in this loop pattern is due to $\Delta\widetilde{D}[k+1]$ lagging behind the $\widetilde{I}[k]$ time series.

Regions	λ_I	θ_I	λ_R	θ_R	λ_D	θ_D
Italy	2.8741	0.4193	2.0701	0.0719	0.1787	0.6750
Spain	1.0803	0.1632	1.2673	0.0553	0.1713	0.6574
Wuhan	0.4661	0.0186	2.6485	0.1377	0.0681	0.0154
Turkey	0.6803	0.0264	2.6537	0.1763	0.0135	0.0146
Hubei	0.1745	0.0075	2.2779	0.1866	0.0033	0.0015
Romania	0.3902	0.0204	0.0026	0.0008	0.0632	0.8985
Germany	0.4122	0.0230	0.0028	0.0007	0.1878	0.1371
Denmark	1.0399	0.0387	0.8382	0.4240	0.0081	0.0045

Table S1. Inferred parameters of the reporting delays under the assumption of the Pólya-Aeppli distribution.

Country/Region	Before, $O_b(\widetilde{Y})$	After, $O_b(Y)$	Relative change, $(O_b(Y) - O_b(\widetilde{Y}))/O_b(\widetilde{Y})$
Italy	0.24	0.84	2.5
Spain	0.36	0.98	1.7
Wuhan	0.06	0.63	9.5
Turkey	0.26	0.85	2.5
Hubei	0.10	0.71	6.1
Romania	0.36	0.70	0.94
Germany	0.37	0.76	1.1
Denmark	0.31	0.72	1.3

Table S2. Relative changes in the objective function $O_b(\widetilde{Y})$ before and after the removal of reporting delays.

C. Forecast the COVID-19 pandemic. The COVID-19 pandemic is predicted based on the Algorithms 1 and 2. Algorithm 1 is to forecast the future infected fractions without considering the effect of reporting delays. Algorithm 2 is to forecast the future infected fractions considering the reporting delays.

Algorithm 1 `Epidemic forecast without removal of reporting delays, experiment 1.`

Input: fraction of daily reported infected cases $\Delta\tilde{I}[1], ..., \Delta\tilde{I}[n]$.
Output: predicted fraction of infections $\Delta\hat{I}[n+1], ..., \Delta\hat{I}[n+n_{\text{pred}}]$.
Smooth the data by Matlab toolbox *smoothdata*.
Set the initial value of the loss function $\Theta_s \leftarrow 1$; the initial infection rate $\beta_s \leftarrow 0.01$; the initial removed rate (the sum of recovered rate and deceased rate) $\gamma_s \leftarrow 0.01$; the initial infected fraction $I_s[0] \leftarrow 0.01$;
for $\beta = 0.01, 0.02, ..., 1$ **do**
  for $\gamma = 0.01, 0.02, ..., 1$ **do**
    for $k = 0, 1, ..., 100$ **do**
      $I[0] = 10^{-2-k/25}$;
      Numerically solve the equations of the SIRD model based on the parameters $I[0]$, β and γ and obtain an infection curve $\Delta\mathcal{I}[1], ..., \Delta\mathcal{I}[n+n_p]$.
      Scale the simulated curve $\Delta\mathcal{I}[1], ..., \Delta\mathcal{I}[n+n_p]$ to data $\Delta\hat{I}[1], ..., \Delta\hat{I}[n+n_p]$ by letting $\Delta\hat{I}[i] = \Delta\mathcal{I}[i] \times \Delta\tilde{I}[1]/\Delta\mathcal{I}[1]$ for day $i - 1, 2, \cdots, n+n_p$.
      Calculate the lose function $\Theta = \sqrt{\frac{1}{n}\sum_{k=0}^{n-1}(\Delta\hat{I}[k] - \Delta\tilde{I}[k])^2}$.
      if $\Theta < \Theta_s$ **then**
        $\Theta_s \leftarrow \Theta$; $I_s[0] \leftarrow I[0]$; $\beta_s \leftarrow \beta$; $\gamma_s \leftarrow \gamma$.
Obtain the best forecast results based on the optimized parameters $I_s[0]$, β_s and γ_s.

Algorithm 2 `Forecast the real pandemic considering the reporting delays`

Input: fraction of daily reported infected cases $\Delta\tilde{I}[1], ..., \Delta\tilde{I}[n]$; fraction of daily reported deceased cases $\Delta\tilde{D}[1], ..., \Delta\tilde{D}[n]$; fraction of daily reported recovered cases $\Delta\tilde{R}[1], ..., \Delta\tilde{R}[n]$; prediction time n_{pred}.
Output: predicted fraction of infections $\Delta\hat{I}[n+1], ..., \Delta\hat{I}[n+n_{\text{pred}}]$.
Smooth the data by Matlab toolbox *smoothdata*.
Infer delay distribution for infected cases T_I using the reported data $\Delta\tilde{I}$, $\Delta\tilde{R}$ and $\Delta\tilde{D}$.
Obtain the inferred data $\Delta\bar{I}[1], ..., \Delta\bar{I}[n]$ by removing the effect of reporting delays.
Set the initial value of the loss function $\Theta_s \leftarrow 1$; the initial infection rate $\beta_s \leftarrow 0.01$; the initial removed rate (the sum of recovered rate and deceased rate) $\gamma_s \leftarrow 0.01$; the initial infected fraction $I_s[0] \leftarrow 0.01$;
for $\beta = 0.01, 0.02, ..., 1$ **do**
  for $\gamma = 0.01, 0.02, ..., 1$ **do**
    for $k = 0, 1, ..., 100$ **do**
      $I[0] = 10^{-2-k/25}$;
      Numerically solve the equations of SIR model based on the parameters $I[0]$, β and γ and obtain an infection curve $\Delta\mathcal{I}[1], ..., \Delta\mathcal{I}[n+n_p]$.
      Scale the simulated curve $\Delta\mathcal{I}[1], ..., \Delta\mathcal{I}[n+n_p]$ to data $\Delta\hat{I}[1], ..., \Delta\hat{I}[n+n_p]$ by letting $\Delta\hat{I}[i] = \Delta\mathcal{I}[i] \times \Delta\bar{I}[1]/\Delta\mathcal{I}[1]$ for day $i - 1, 2, \cdots, n+n_p$.
      Calculate the lose function $\Theta = \sqrt{\frac{1}{n}\sum_{k=0}^{n-1}(\Delta\hat{I}[k] - \Delta\bar{I}[k])^2}$.
      if $\Theta < \Theta_s$ **then**
        $\Theta_s \leftarrow \Theta$; $I_s[0] \leftarrow I[0]$; $\beta_s \leftarrow \beta$; $\gamma_s \leftarrow \gamma$.
Obtain the best forecast results based on the optimized parameters $I_s[0]$, β_s and γ_s.
Add the reporting delays to the forecast data using Eq. [1]

D. Governing equation of the time shift. The fraction $Y[k]$ is contained in $[0,1]$ and measured per day. Hence, $Y[k]$ is a real-valued random variable and the analysis is in discrete time k, where k is an integer that represents day $k - k_0$ since the start of the measurements of the fraction Y at day $k_0 \in \mathbb{Z}$. In the sequel, as in (1), capital letter refer to random variable and small letters to numbers.

Our basic observation lies in a time delay T between the *real* value $Y[k]$ and the reported value $Y_{\text{rep}}[k]$, which translates to

$$Y[k] = Y_{\text{rep}}[k+T] \quad [1]$$

Here, we implicitly assume that the reported value $Y_{\text{rep}}[k]$ exactly measures the real fraction and only differs from reality in the time delay T. If errors or uncertainties appear, then we can replace $Y_{\text{rep}}[k]$ by $Y_{\text{rep}}[k] = \widetilde{Y}_{\text{rep}}[k] + U[k]$, where U reflects

the uncertainty or error in reporting or measuring. The delay T must be non-negative, i.e. $T \geq 0$, because the real event $Y[k]$ occurs at discrete time k and its reporting occurs at $k + T \geq k$, which cannot be earlier than the time k. Moreover, the delay T is also an integer because we assume a discrete-time analysis, else Eq. [**??**] demands us to take the integer value $[T]$ of $T = [T] + \langle T \rangle$, where the fractional part $0 \leq \langle T \rangle < 1$. We avoid that complication and consider T as a discrete random variable. If T is discrete, then all involved random variables are discrete. Before proceeding, we thus approximate $Y(t)$ at continuous time $k - 1 < t \leq k$, by $Y[k] = \int_{k-1}^{k} Y(u)\,du$. Hence, the instantaneous fraction $Y(t)$, denoted by "round" bracket (.), and cumulative fraction $Y[k]$ in one timeslot, denoted by "square" brackets [.], are physically different random variables! Furthermore, the mean-field approximation (as in Eq. [1] of the main text) only writes the equations for the *average* fraction of infected nodes and we refer to (1, Sec. 17.3.2; Sec. 17.4) for the relation between the Markov process and its mean-field approximation.

The basic observation in [**??**] contains two intertwined random variables, namely the reported fraction Y_{rep} and the delay T. Hence, we use the law of total probability (1, p. 23) and conditioning,

$$\Pr[Y_{\text{rep}}[k+T] \leq y] = \sum_{m=0}^{\infty} \Pr[Y_{\text{rep}}[k+T] \leq y | T = m] \Pr[T = m]$$

Since we confine to a mean-field analysis and are only interested in the *average* fraction of infected (see eq. (1,2) in the main text), we better take the expectation operator $E[.]$ instead of the probability $\Pr[.]$ operator:

$$E[Y_{\text{rep}}[k+T]] = \sum_{m=0}^{\infty} E[Y_{\text{rep}}[k+T] | T = m] \Pr[T = m] \tag{2}$$

which is readily obtained from the former equation by using the definition of the mean (e.g. (1, (2.36) on p. 18)), namely $E[Y_{\text{rep}}[k+T]] = \int_0^1 \Pr[Y_{\text{rep}}[k+T] > y]\,dy$.

This last equation (**??**) involves the conditional expectations $E[Y_{\text{rep}}[k+T] | T = m]$. If we assume that T is independent of Y_{rep}, then $E[Y_{\text{rep}}[k+T] | T = m] = E[Y_{\text{rep}}[k+m]]$. After taking the expectation of the hypothesis [**??**] and combining with [**??**], assuming independence between fraction Y and delay T, then we obtain

$$E[Y[k]] = \sum_{m=0}^{\infty} E[Y_{\text{rep}}[k+m]] \Pr[T = m]$$

Finally, if Y reflects the fraction of infected (and similarly for the other compartments in epidemic models), then we simplify the notation as $I[k] = E[Y[k]]$ and arrive at our approximative observation hypothesis for the average fraction of infected individuals

$$I[k] = \sum_{m=0}^{\infty} I_{\text{rep}}[k+m] \Pr[T = m] \tag{3}$$

In summary, if the time delay T and the reported value Y_{rep} are independent, then the observation hypothesis [**??**] translates the average fraction of infected (similarly for removed and deceased) to the weighted sum of the average fraction of the reported, where the weight is the probability $\Pr[T = m]$ that the (integer) delay T equals m. If independence does not hold, the above derivation shows that it is difficult to compute the relation between $Y_{\text{rep}}[k]$ and $Y[k]$, unless the conditional probabilities in [**??**] can be determined. We argue that independence between T and Y_{rep} is reasonable, although both random variables are weakly positively correlated. Indeed, the larger the fraction Y, the more people need to be checked and the longer the reporting may take. If the checking capacity is sufficiently large, we may assume approximate independence.

D.1. SIR compartments. The discrete-time SIR model is defined (see e.g. (2)), for every node i in the graph G at discrete time k, by viral state vector $v_i[k] = (S_i[k], I_i[k], R_i[k])$. The governing SIR equations in discrete time as

$$I_i[k+1] = (1-\delta_i)I_i[k] + (1 - I_i[k] - R_i[k]) \sum_{n=1}^{N} \beta_{in} I_n[k] \tag{4}$$

$$R_i[k+1] = R_i[k] + \delta_i I_i[k] \tag{5}$$

and the fraction of susceptible individuals follows from the conservation law as

$$S_i[k] = 1 - I_i[k] - R_i[k]$$

Here, β_{in} denotes the infection probability from node i to node n and δ_i is the curing probability of node i. With the initial condition $R_i[0] = 0$, the linear difference equation (**??**) is readily solved for $k > 0$

$$R_i[k] = \delta_i \sum_{m=0}^{k-1} I_i[m]$$

and (**??**) reduces to

$$I_i[k+1] - (1-\delta_i)I_i[k] = (1 - I_i[k] - \delta_i \sum_{m=0}^{k-1} I_i[m]) \left(\beta_{ii} I_i[k] + \sum_{n=1; n\neq i}^{N} \beta_{in} I_n[k] \right) \tag{6}$$

D.2. The time delays T_S, T_I and T_S are correlated. Relation [??] holds for any fraction Y. If we confine to the SIR model with three compartments, then the approximate equations [??] are

$$\begin{cases} S[k] = \sum_{m=0}^{\infty} S_{\text{rep}}\left[k+m\right] \Pr\left[T_S = m\right] \\ I[k] = \sum_{m=0}^{\infty} I_{\text{rep}}\left[k+m\right] \Pr\left[T_I = m\right] \\ R[k] = \sum_{m=0}^{\infty} R_{\text{rep}}\left[k+m\right] \Pr\left[T_R = m\right] \end{cases}$$

The compartmental conservation law $S\left[k\right] + I\left[k\right] + R\left[k\right] = 1$ (and also $S_{\text{rep}}\left[k\right] + I_{\text{rep}}\left[k\right] + R_{\text{rep}}\left[k\right] = 1$) leads to

$$\begin{aligned} 1 &= \sum_{m=0}^{\infty} \left(1 - I_{\text{rep}}\left[k+m\right] - R_{\text{rep}}\left[k+m\right]\right) \Pr\left[T_S = m\right] + \sum_{m=0}^{\infty} I_{\text{rep}}\left[k+m\right] \Pr\left[T_I = m\right] \\ &\quad + \sum_{m=0}^{\infty} R_{\text{rep}}\left[k+m\right] \Pr\left[T_R = m\right] \end{aligned}$$

or

$$\sum_{m=0}^{\infty} I_{\text{rep}}\left[k+m\right]\left(\Pr\left[T_S = m\right] - \Pr\left[T_I = m\right]\right) = \sum_{m=0}^{\infty} R_{\text{rep}}\left[k+m\right]\left(\Pr\left[T_R = m\right] - \Pr\left[T_S = m\right]\right)$$

This relation illustrates that the timeshifts T_S, T_I and T_R are correlated. The correlation or dependence is expected because the timeshifts T_S, T_I and T_R are all obtained from a same measurement or reporting procedure. Interestingly, if we assume that T_S, T_I and T_R have the same distribution, $\Pr\left[T_S = m\right] = \Pr\left[T_I = m\right] = \Pr\left[T_R = m\right]$, (which does not imply that the timeshifts, i.e. the random variables, are the same!, i.e. $T_S \neq T_I \neq T_R$), then the compartmental conservation is always satisfied.

D.3. Set of quadratic equations for $\Pr\left[T_I = m\right]$. Introducing the observation hypothesis $I_i[k] = \sum_{m=0}^{\infty} I_{\text{rep,i}}\left[k+m\right] \Pr\left[T_{I,i} = m\right]$ in [??] at node i into [??] yields

$$\begin{aligned} Q_L\left[k\right] &= \sum_{m=0}^{\infty} \left(I_{\text{rep,i}}\left[k+1+m\right] - (1-\delta_i) I_{\text{rep,i}}\left[k+m\right]\right) \Pr\left[T_{I,i} = m\right] \\ Q_R\left[k\right] &= \left(1 - \sum_{m=0}^{\infty} \left\{ I_{\text{rep,i}}\left[k+m\right] - \delta_i \sum_{l=0}^{k-1} I_{\text{rep,i}}\left[l+m\right] \right\} \Pr\left[T_{I,i} = m\right]\right) \\ &\quad \left(\beta_{ii} \sum_{m=0}^{\infty} I_{\text{rep,i}}\left[k+m\right] \Pr\left[T_{I,i} = m\right] + \sum_{n=1;n\neq i}^{N} \beta_{in} \sum_{m=0}^{\infty} I_{\text{rep,n}}\left[k+m\right] \Pr\left[T_{I,n} = m\right]\right) \end{aligned}$$

where the left-hand side Q_L and right-hand side Q_R of equation (??) are equal, i.e. $Q_L\left[k\right] = Q_R\left[k\right]$. We omit in the sequel the index R and L. We can proceed if we assume that the time-delay in different nodes has the same distribution, i.e. $\Pr\left[T_{I,i} = m\right] = \Pr\left[T_I = m\right]$ for each node i in the contact graph. Then,

$$\begin{aligned} Q\left[k\right] &= \sum_{m=0}^{\infty} \left(I_{\text{rep,i}}\left[k+1+m\right] - (1-\delta_i) I_{\text{rep,i}}\left[k+m\right]\right) \Pr\left[T_I = m\right] \\ &= \sum_{m=0}^{\infty} \left\{ 1 - I_{\text{rep,i}}\left[k+m\right] - \delta_i \sum_{l=0}^{k-1} I_{\text{rep,i}}\left[l+m\right] \right\} \Pr\left[T_I = m\right] \sum_{r=0}^{\infty} \left(\sum_{n=1}^{N} \beta_{in} I_{\text{rep,n}}\left[k+r\right]\right) \Pr\left[T_I = r\right] \end{aligned}$$

We rewrite the double sum

$$\begin{aligned} \sum_{m=0}^{\infty} \sum_{r=0}^{\infty} f\left(m\right) g\left(r\right) &= \sum_{m=0}^{\infty} \sum_{r=0}^{\infty} \left\{ 1 - I_{\text{rep,i}}\left[k+m\right] - \delta_i \sum_{l=0}^{k-1} I_{\text{rep,i}}\left[l+m\right] \right\} \left(\sum_{n=1}^{N} \beta_{in} I_{\text{rep,n}}\left[k+r\right]\right) \\ &\quad \times \Pr\left[T_I = m\right] \Pr\left[T_I = r\right] \end{aligned}$$

as

$$\sum_{m=0}^{\infty} \sum_{r=0}^{\infty} f\left(m\right) g\left(r\right) = \sum_{m=0}^{\infty} f\left(m\right) \sum_{r=0}^{m-1} g\left(r\right) + \sum_{m=0}^{\infty} f\left(m\right) \sum_{r=m}^{\infty} g\left(r\right)$$

Reversing the summations in the last sum,

$$\sum_{m=0}^{\infty} f\left(m\right) \sum_{r=m}^{\infty} g\left(r\right) = \sum_{r=0}^{\infty} g\left(r\right) \sum_{m=0}^{r} f\left(m\right) = \sum_{r=0}^{\infty} g\left(r\right) f\left(r\right) + \sum_{r=0}^{\infty} g\left(r\right) \sum_{m=0}^{r-1} f\left(m\right)$$

Hence, we obtain (after interchanging the indices in the last)

$$\sum_{m=0}^{\infty}\sum_{r=0}^{\infty} f(m)\,g(r) = \sum_{r=0}^{\infty} g(r)\,f(r) + \sum_{m=0}^{\infty}\sum_{r=0}^{m-1}\{f(m)\,g(r) + g(m)\,f(r)\}$$

Finally, for each discrete time $0 \leq k$, we obtain

$$\begin{aligned} Q[k] &= \sum_{m=0}^{\infty}\left(I_{\text{rep,i}}[k+1+m] - (1-\delta_i)I_{\text{rep,i}}[k+m]\right)\Pr[T_I = m] \\ &= \sum_{m=0}^{\infty}\left(\sum_{n=1}^{N}\beta_{in}I_{\text{rep,n}}[k+m]\right)\left(1 - I_{\text{rep,i}}[k+m] - \delta_i\sum_{l=0}^{k-1}I_{\text{rep,i}}[l+m]\right)\left(\Pr[T_I = m]\right)^2 \\ &\quad + \sum_{m=0}^{\infty}\Pr[T_I = m]\sum_{r=0}^{m-1}\Pr[T_I = r]\left\{\begin{array}{l} \sum_{n=1}^{N}\beta_{in}I_{\text{rep,n}}[k+r]\left(1 - I_{\text{rep,i}}[k+m] - \delta_i\sum_{l=0}^{k-1}I_{\text{rep,i}}[l+m]\right) \\ +\sum_{n=1}^{N}\beta_{in}I_{\text{rep,n}}[k+m]\left(1 - I_{\text{rep,i}}[k+r] - \delta_i\sum_{l=0}^{k-1}I_{\text{rep,i}}[l+r]\right) \end{array}\right\} \end{aligned}$$

In order to limit the infinite sum, we can choose a time point M and assume that $\Pr[T_I = m] = 0$ for $m > M$. Denoting $x[m] = \Pr[T_I = m]$, the above equation possesses the form

$$\sum_{m=0}^{M} A_m[k]\,x[m] = \sum_{m=0}^{M} B_m[k]\,C_m[k]\,(x[m])^2 + \sum_{m=0}^{M} x[m]\sum_{r=0}^{m-1} x[r]\,\{B_r[k]\,C_m[k] + B_m[k]\,C_r[k]\}$$

which leads to a set of quadratic equations in the variables $\{x[m]\}_{0\leq m\leq M}$ for each $k \geq 0$, that can be solved numerically, together with $\sum_{m=0}^{M} x[m] = 1$.

In summary, assuming an SIR epidemics with the knowledge of the infection probabilities β_{in} between node i and n and the nodal curing probability δ_i at each node i, then the set $\{I_{\text{rep,n}}[k]\}_{k\geq k_0}$ of reported fractions of infected $I_{\text{rep,n}}[k]$ at discrete time k and at each node n is sufficient to compute the delay distribution $\Pr[T_I = m]$ (where we have assumed that the distribution $\Pr[T_{I,n} = m] = \Pr[T_I = m]$ is the same for each node). The resulting set of quadratic equation is rather demanding to solve and justifies the approach in the main text: we assume that, within a broad class of probability distributions with two parameters, a certain range of those parameters satisfies the quadratic set for $\Pr[T_I = m]$.

References

1. P. Van Mieghem. *Performance Analysis of Complex Networks and Systems*. Cambridge University Press, Cambridge, U.K., 2014.
2. B. Prasse, M. A. Achterberg, L. Ma, and P. Van Mieghem. Network inference-based prediction of the COVID-19 outbreak in the Chinese province Hubei. *Applied Network Science*, to appear, also on arXiv:2002.04482 2020.
3. P. Van Mieghem. The asymptotic behaviour of queueing systems: Large deviations theory and dominant pole approximation. *Queueing Systems*, 23:27–55, 1996.
4. E. C. Titchmarsh. *The Theory of Functions*. Oxford University Press, Amen House, London, 1964.

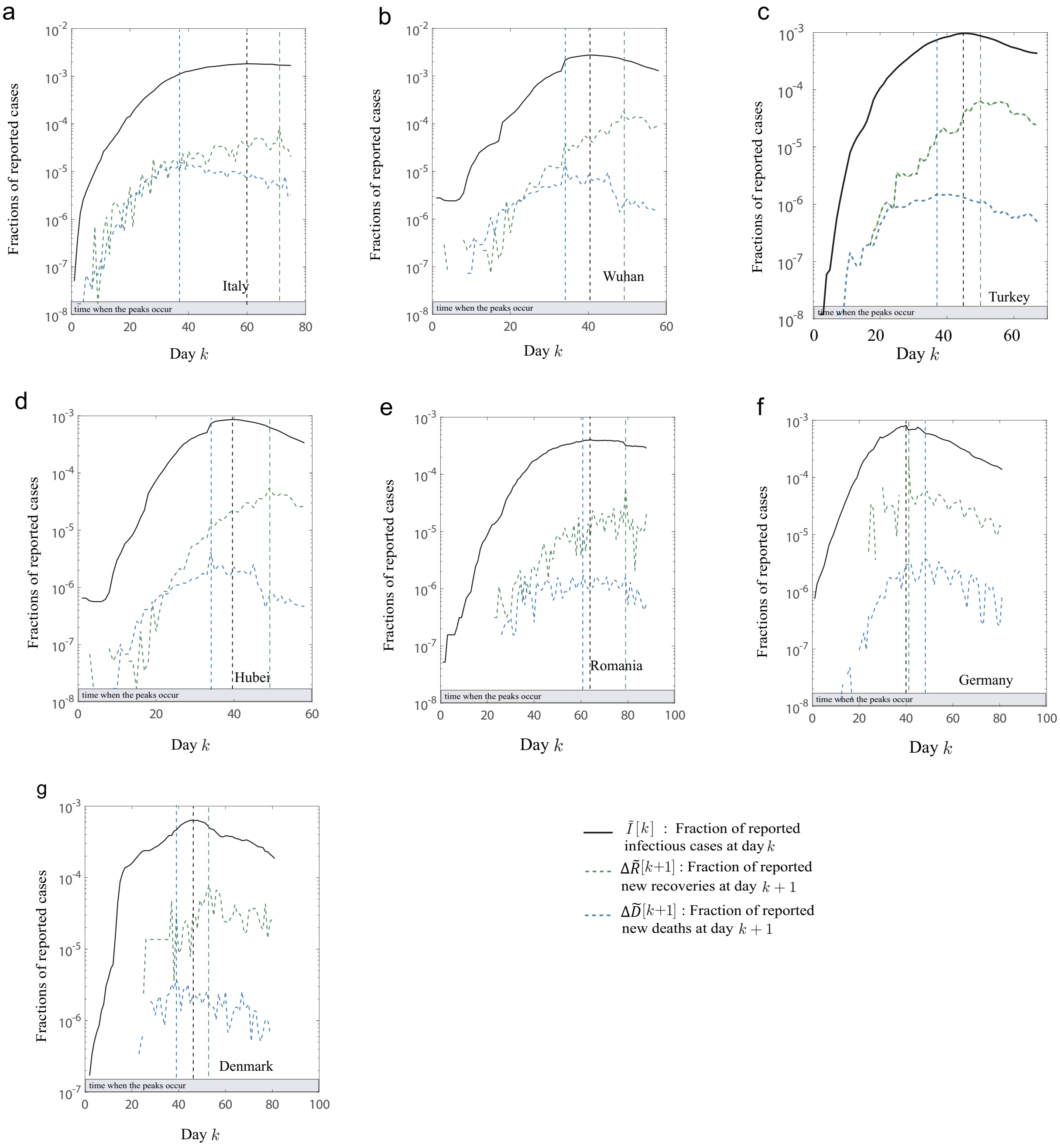

Fig. S1. Time series of fractions of reported active infections $\tilde{I}[k]$, new reported recoveries $\Delta\widetilde{R}[k+1]$ and new reported deaths $\Delta\widetilde{D}[k+1]$ for (a) Italy, (b) Wuhan province, (c) Turkey, (d) Hubei province, (e) Romania, (f) Germany, and (g) Denmark. Vertical dashed lines highlight the locations of data peaks.

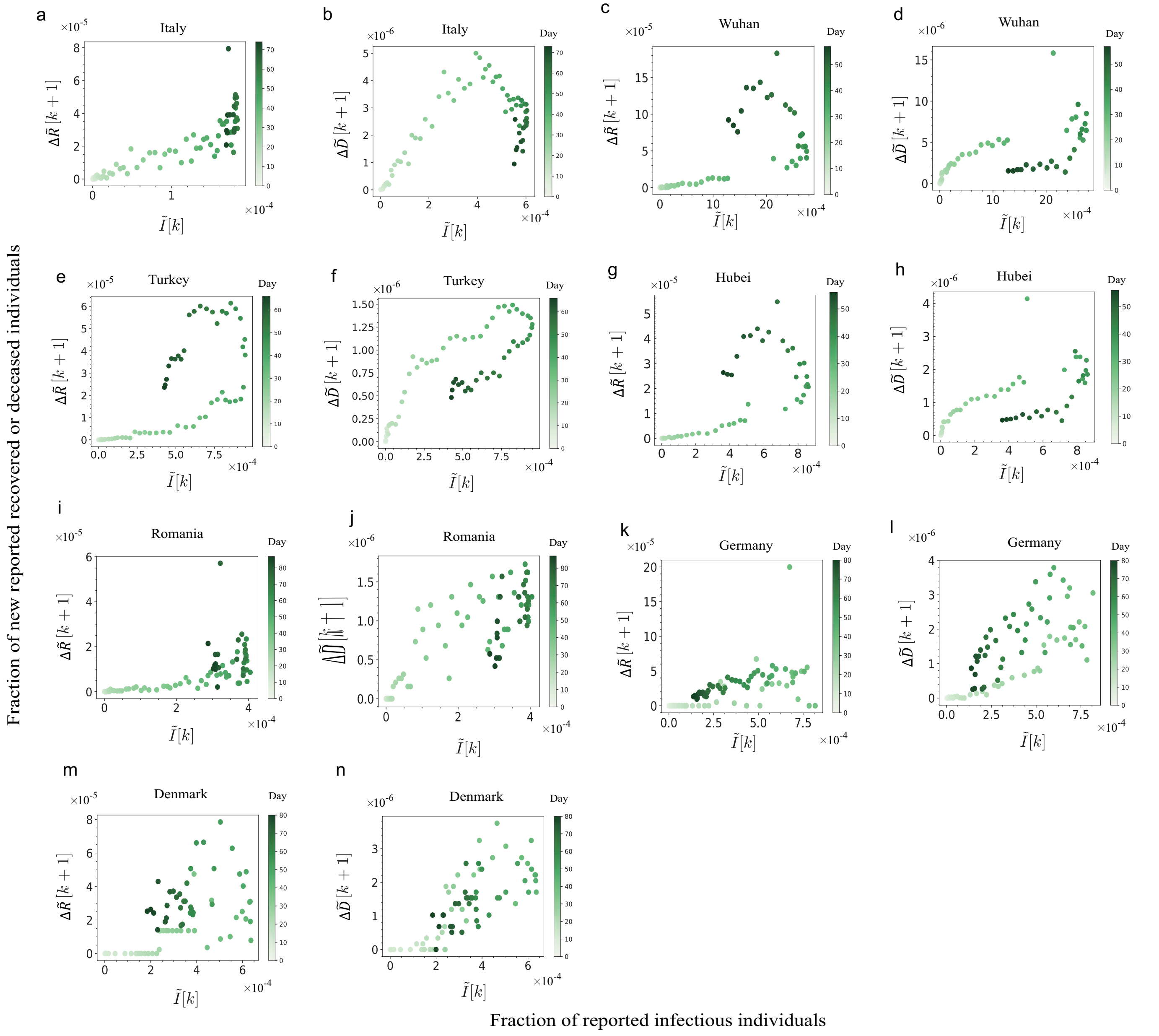

Fig. S2. Panels display pairwise color-coded scatter plots of the fraction of reported active infections $\tilde{I}[k]$ as a function of the fraction of new reported recoveries $\Delta\widetilde{R}[k+1]$ and the fraction of reported active infections $\tilde{I}[k]$ as a function of the fraction of reported deaths $\Delta\widetilde{D}[k+1]$ for (a,b) Italy, (c,d) Wuhan province, (e,f) Turkey, (g,h) Hubei province, (i,j) Romania, (k,l) Germany, and (m,n) Denmark. Colors, from light to dark green, reflect different days in the data ranging, respectively, from $k=0$ to $k=57$. Note that data points for $\tilde{I}[k]$ versus $\Delta\widetilde{R}[k+1]$ evolve in the counter-clockwise direction, while data points for $\tilde{I}[k]$ versus $\Delta\widetilde{D}[k+1]$ evolve in the clockwise direction.

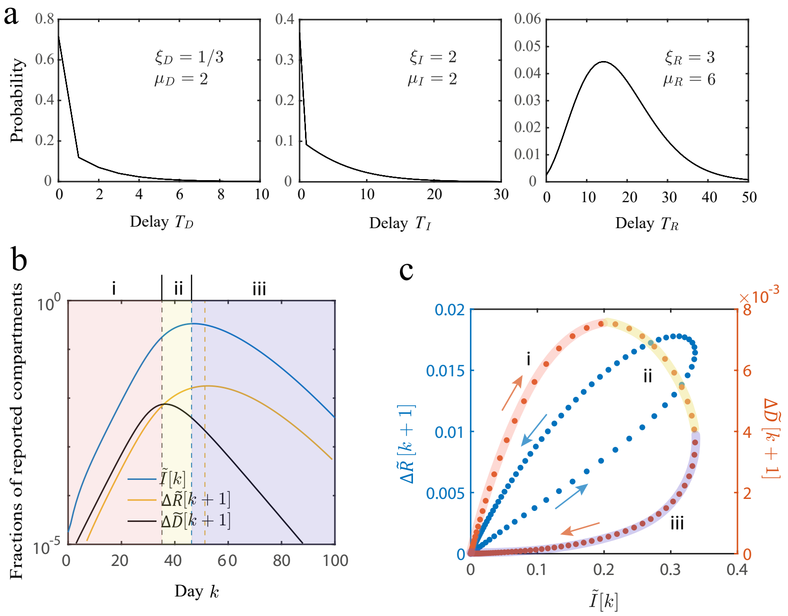

Fig. S3. Effects of reporting delays on synthetic data. (a) Pólya-Aeppli distributions generating reporting delays in the deceased, infected and recovered datasets. (b) Epidemic data generated with the SIRD model containing reporting delays. (c) Changes in the fraction of recovered $\Delta R[k+1]$ and deceased $\Delta D[k+1]$ individuals as a function of the fraction of infected individuals $I[k]$. Note that reporting delays lead to the appearance of the clockwise and counterclockwise loop patterns in the epidemic data.

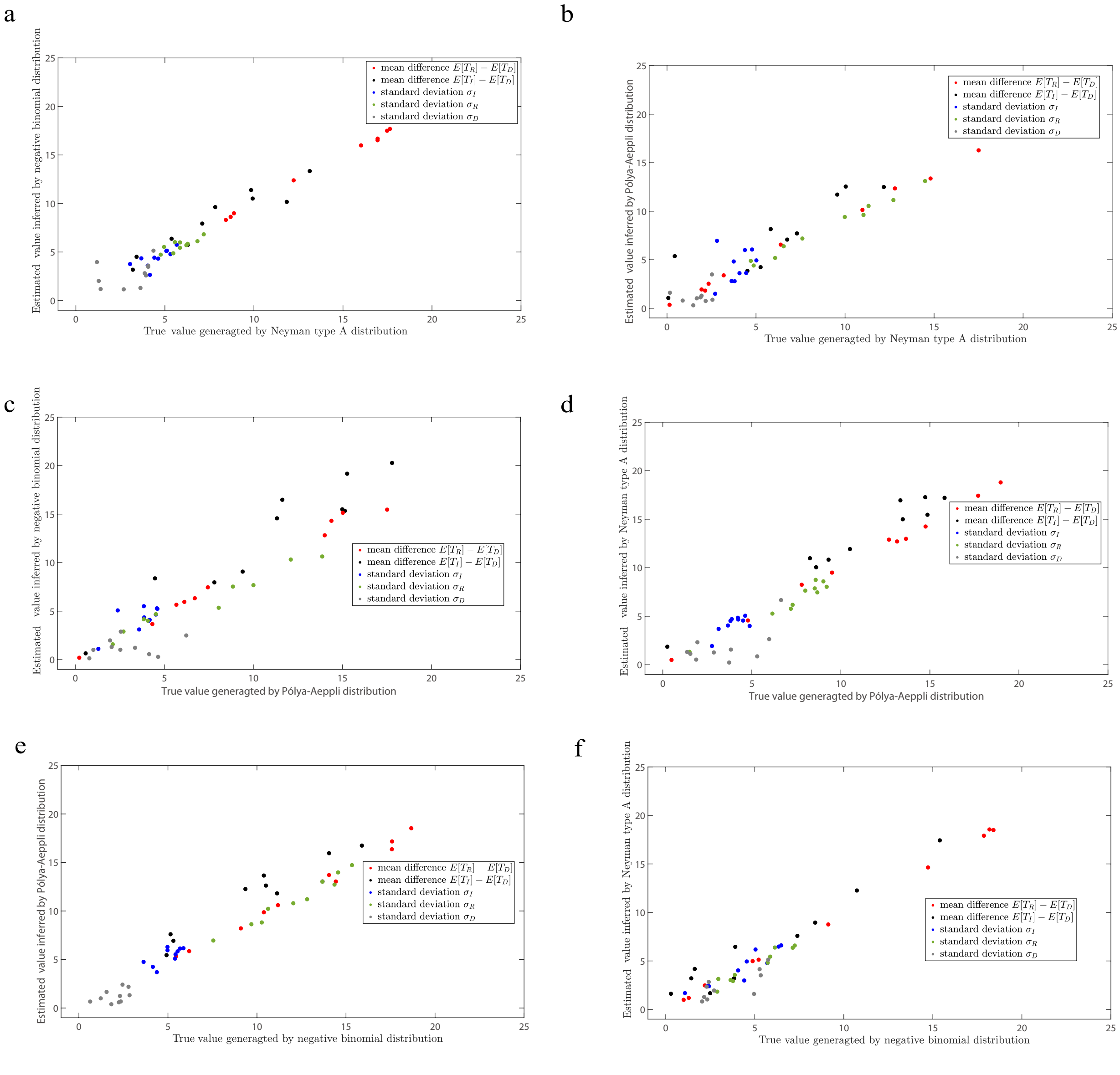

Fig. S4. Inference of reporting delays on synthetic data. This figure will feature 6 panels corresponding to all pairs of distributions: data generated with X, inferred with Y.

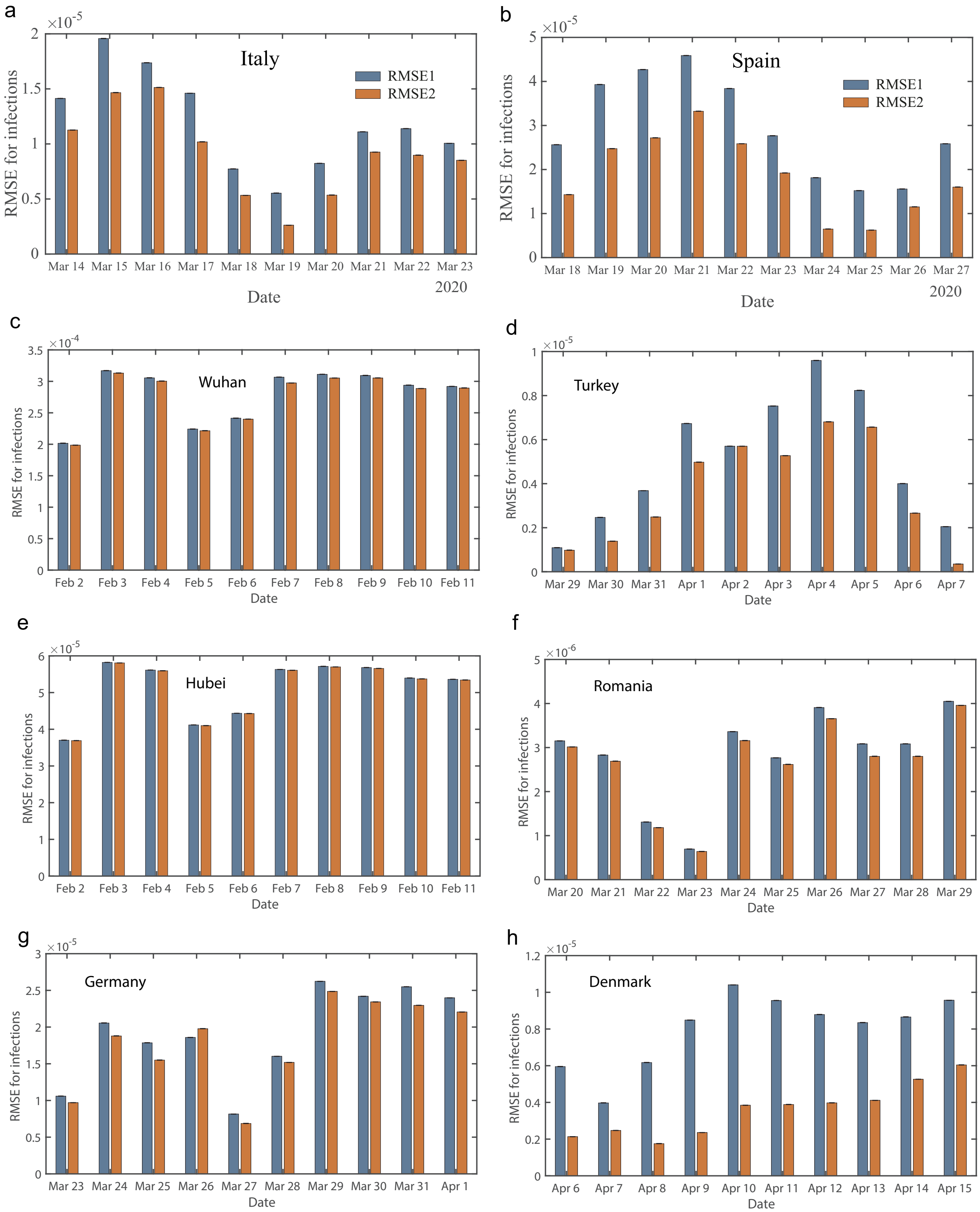

Fig. S5. Forecasts of the COVID-19 pandemic using the SIRD model with and without accounting for reporting delays. Each panel shows the RMSE between the forecast results and the real reported data when we forecast the future prevalence on different dates.

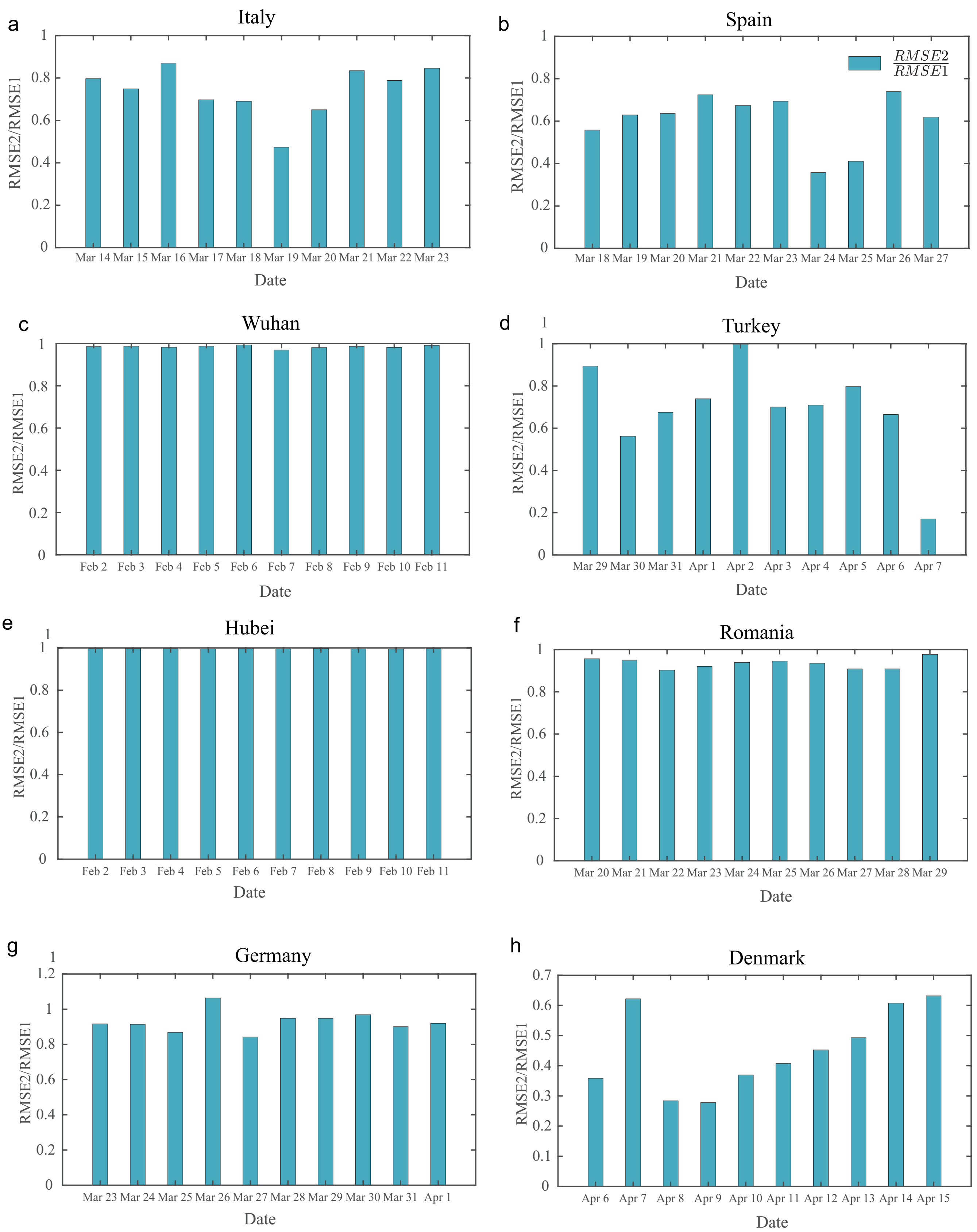

Fig. S6. The ratio between RMSE2 and RMSE1 for all 8 regions. Note that the forecast improvements are more significant for Spain, Italy, Turkey and Denmark than for the other regions.
